# Novel Fitc-Labeled Igy Antibody: Fluorescence Imaging *Toxoplasma Gondii In Vitro*

**DOI:** 10.1038/s41598-017-00930-1

**Published:** 2017-04-12

**Authors:** Mehtap Sert, Rabia Cakir Koc, Yasemin Budama Kilinc

**Affiliations:** Department of Bioengineering, Faculty of Chemical and Metallurgical Engineering, Yildiz Technical University, 34210 Esenler-Istanbul, Turkey

## Abstract

Toxoplasmosis is caused by *T*. *gondii* and can create serious health problems in humans and also worldwide economic harm. Because of the clinical and veterinary importance of toxoplasmosis, its timely and accurate diagnosis has a major impact on disease-fighting strategies. *T*. *gondii* surface antigen 1 (SAG1), an immunodominant-specific antigen, is often used as a diagnostic tool. Therefore, the aim of this study was the optimization of novel fluorescein isothiocyanate (FITC) labeling of the SAG1-specific IgY antibody to show the potential for immunofluorescence imaging of *T*. *gondii in vitro*. The specificity of IgY antibodies was controlled by an enzyme-linked immunosorbent assay (ELISA), and the concentration of the IgY antibody was detected using a spectrophotometer. The optimum incubation time and FITC concentration were determined with a fluorescence spectrometer. The obtained FITC-labeled IgY was used for marking *T*. *gondii* tachyzoites, which were cultured *in vitro* and viewed using light microscopy. The interaction of the fluorescence-labeled antibody and the *T*. *gondii* tachyzoites was examined with a fluorescence microscope. In this study, for the first time, a FITC-labeled anti-SAG1 IgY antibody was developed according to ELISA, fluorescence spectrometer, and fluorescence imaging of cell culture. In the future, the obtained FITC-labeled *T*. *gondii* tachyzoites’ specific IgY antibodies may be used as diagnostic tools for the detection of *T*. *gondii* infections in different samples.

## Introduction

*Toxoplasma gondii* is an obligate intracellular parasite that infects approximately 30% of the world’s population^1^. The toxoplasmosis disease is caused by *T*. *gondii* and produces health problems in humans as well as economic harm worldwide. The condition is generally asymptomatic, but it can cause local lymphadenopathy, mortality in immunodeficient patients^2^ and hydrocephalus, intracranial calcification, chorioretinitis, fever, anemia, hypothermia, microcephaly, microphthalmia, and deafness in newborns. Congenital toxoplasmosis can also cause spontaneous abortion, premature birth, and stillbirth in pregnancy^3^. Toxoplasmosis is responsible for economic and health problems not only in humans but also in animals^4–6^, as the disease is widely present in many animal species^5^.

Because of the clinical and veterinary importance of toxoplasmosis, the diagnosis has a major impact on coping strategies of clinicans. Many diagnostic methods exist for the detection of toxoplasmosis (biologic, histological, or some combination of these methods)^5^, with each having its own advantages and disadvantages regarding cost, sensitivity, specificity, ease of use, etc.^7^.

Antibodies are useful tools for many diagnostic tests^8^, especially serological tests. Among antibody production methods, specific yolk antibody production has recently gained importance with its numerous advantages over monoclonal antibody production methods^9^. There are a limited number of studies in the literature about *Toxoplasma*-specific IgY production. Among these, Hassl *et al*. purified eggs to obtain IgY antibodies directed against the soluble proteins of *T*. *gondii* from immunized hens. They compared the specificity of IgY and IgG antibodies obtained from immunized hens and hyper-immunized rabbits, respectively^10^. In another study, Ferreira *et al*. also obtained polyclonal anti-*T*. *gondii* IgY antibodies against soluble tachyzoite antigens from immunized hens, and they highlighted the diagnostic applications of polyclonal IgY antibodies^11^. In one of the most recent studies, SAG1-specific antibodies were obtained from immunized hens^12^.

However, in the literature, there is no study of the development of fluorescent-labeled specific IgY antibodies against the surface antigen of *T*. *gondii* (SAG1) or the application of a direct immunofluorescence assay. Therefore, the aim of this study was the optimization of the fluorescent dye-labeling procedure between the SAG1-specific IgY antibody and FITC to develop a novel dye to detect *T*. *gondii* parasites.

## Materials

Phosphate-buffered saline (PBS) and polyethylene glycol (PEG) 6000 (Santa Cruz Biotechnology, Inc., Santa Cruz, CA, USA) were used to purify the eggs. Na_2_HPO_4_(7 H_2_O) (Merck, Kenilworth, NJ, USA), NaH_2_PO_4_(2 H_2_O) (Duchefa Biochemie B.V., Haarlem, Netherlands), NaCl (Duchefa Biochemie B.V.), HCl (Merck), and NaOH (Merck) were utilized for the PBS buffer. Na_2_CO_3_ (Sigma-Aldrich, St. Louis, MO, USA) and HCl (Merck) or NaOH (Merck) were used to prepare the sodium carbonate buffer. PBS buffer, coupling buffer, PBS-T solution and blocking buffer were used for the ELISA. PBS buffer for the ELISA was formed from Na_2_HPO_4_ (12 H_2_O) (Merck), (KH_2_PO_4_) (Merck), KCl (Merck), HCl (Merck), NaOH (Merck), and NaCl (Duchefa Biochemie B.V.). The coupling solution was made from Na_2_CO_3_ (Sigma-Aldrich), NaHCO_3_ (Merck), NaN_3_ (Merck), HCl (Merck), and NaOH (Merck). Na_2_HPO_4_ (7H_2_O) (Merck), NaH_2_PO_4_(2H_2_O) (Duchefa Biochemie B.V.), NaCl (Duchefa Biochemie B.V.), HCl (Merck), NaOH (Merck), and Tween-20 (Merck) were used for the PBS-T solution. Also, BSA (Sigma-Aldrich) was added to the PBS for the blocking buffer. P-nitrophenyl was provided by Sigma-Aldrich. Fluorescein 5-isothiocyanate (FITC, SIGMA7250), dimethylsulfoxide (DMSO), and ethanolamine were obtained from Sigma-Aldrich.

### Fluorescence Measurements

Fluorescence emission spectra were obtained using a QM-4/2003 Quanta Master Steady State Spectrofluorimeter (Photon Technology International, London, ON, CAN) operating in quanta-counting mode. The slits of the excitation and emission monochromators were adjusted to 2 nm. The excitation was performed at 280 and 495 nm. Emission spectrums was obtained at 324 nm and 520 nm, respectively.

## Methods

### Production of the IgY Antibody

Lohmann Brown hens that were 20 weeks old were immunized with 20 µg of recombinant SAG1 three times and 10 µg of rSAG1 one time every two weeks. Eggs that contained the highest level of the antibody were given booster doses and used in the study^12^. Control eggs were obtained from hens injected with phosphate buffered saline (PBS). The egg yolk antibody was isolated according to Polson *et al*.’s precipitation procedure. Briefly, the eggs were first cleaned with ethanol. The precipitation method followed; polyethylene glycol 6000 (PEG 6000; Sigma-Aldrich, St. Louis, MO, US) was used three times, and dialysis was then applied to the eggs to purify the IgY antibodies^13^. PBS, 3.5% PEG 6000, and the egg yolk were combined and then centrifuged. This procedure was repeated on the obtained supernatant with 8.5% and 12% PEG 6000 so that the pellet containing the IgY antibodies was dialyzed^12^. All of the experiments used in animals were approved by the Animal Ethics Committee of the University of Osmangazi in Eskisehir, Turkey. All methods were carried out in accordance with the ethical standards and guidelines of the responsible committee.

### Protein Activity and Concentration

Protein activity was determined using the enzyme-linked immunosorbent assay (ELISA) method. Briefly, 96-well plates were coated with SAG1 protein and incubated overnight at +4 °C. After the incubation, these plates were washed three times with PBS-T. Then, the plates were coated with the blocking buffer (10 mg/ml bovine serum albumin (BSA) and incubated for 1 h at 37 °C. After the incubation time, three washings were again performed. A 1:100 dilution of the obtained IgY antibody was added to the wells (n = 5) with PBS-T and incubated for 1 hour at 37 °C^12^. The plates were then washed with PBS-T, and alkaline phosphatase conjugated anti-chicken IgY antibody was added at 1:1000 dilutions before the plates were incubated once more. Finally, 100 µl of the substrate solution (SIGMAFAST p-Nitrophenyl phosphate tablets) were added to each well and incubated for 45 min in the dark, and then the absorbance was measured at 405 nm with a microtiter plate reader (Thermo Labsystems Multiskan Ascent 354 Microplate Photometer).

The protein concentration of the purified eggs was calculated with the BioSpec-nano Small-volume UV spectrophotometer (Shimadzu, Kyoto, Japan). The absorbance of the protein solution exceeded 1.4. At 280 nm, the absorbance value of a protein solution should be between 0.2 and 1.4; therefore, the antibody solution was appropriately diluted. The antibody concentration of the diluted samples was measured three times at 280 nm with the spectrophotometer. The diluted protein solution was used in the fluorescence experiments.

### Optimization of the Incubation Time and Concentration of FITC for Labeling with SAG1-specific IgY

The IgY antibody solution was prepared in a 0.1-M sodium carbonate buffer (pH: 9.0). Amine reactive dye FITC was dissolved in dimethyl sulfoxide (DMSO) at a concentration of 1 mg/ml as suggested in the literature^14^ and then was prepared in the buffer stock with a ratio of 1:100. After a single addition of dye from the buffer stock to the antibody solution, the excitation wavelength was adjusted to 495 nm. The measurement intervals were every 1 min. The optimum incubation time was determined as the time when the fluorescence intensity ceased (Fig. 3).

Next, the optimum amounts of FITC concentration for anti-SAG1 IgY antibodies labeled with FITC were studied; a titration experiment was carried out by increasing the concentration from 10 µl to 500 µ with 10-µl intervals of FITC (2 mg/ml IgY antibody in each solution). The reaction was terminated by adding 0.1 M ethanol amine (Sigma-Aldrich). The FITC-labeled anti-SAG1 IgY was incubated at room temperature for 15 min in darkness and stored at −20 °C with BSA (Fig. 4).

### Conjugation of the FITC and IgY Antibodies Obtained from the Control Eggs

The FITC and IgY antibodies (control) were conjugated using the same method as applied for labeling with SAG1-specific IgY. The concentration (400 µl of 10 µg/ml FITC for 2 mg/ml IgY) and incubation time (4 min) were selected according to the results obtained regarding the optimization of the incubation time and the concentration of FITC experiments. The reaction was terminated by adding 0.1 M ethanol amine (Sigma-Aldrich). The FITC-labeled anti-SAG1 IgY was incubated at room temperature for 15 min in dark conditions and then used in experiments termed “Staining of *T*. *gondii* Parasites with FITC-labeled IgY Antibody Obtained from Control Eggs.”

### *In Vitro* Culture of *T*. *gondii* Tachyzoites

*T*. *gondii* tachyzoites were kindly supplied by Dr. Cahit Babür of the Public Health Institution of Turkey. The tachyzoites were washed three times with PBS containing penicillin-streptomycin and then centrifuged for five minutes at 1500 rpm. The supernatant was poured off, and the number of tachyzoites was counted using hemocytometry. The tachyzoites were added to the cultured L929 cells *in vitro* (the ratio of L929 cells to tachyzoites was 1:10) and incubated at 37 °C in 5% CO_2_. The tachyzoites were released from the cells three to five days after the co-culture began. Proliferated tachyzoites were used for direct immunofluorescence assay.

### Identification of *T*. *gondii* by Direct Microscopic Examination and Giemsa Staining

Tachyzoites cultured with L929 were directly observed using an inverted microscope with x40 lens magnification during the culture. After three to five days of culture, the released tachyzoites were collected and placed in a thin layer on microscopic slides. Next, the tachyzoites were fixed with methanol and dried at room temperature. Giemsa stain was added to the slides, which were incubated at room temperature for 15 min. Finally, the prepared slides were washed, and the stained tachyzoites were observed using a light microscope^15^.

### Staining of *T*. *gondii* Parasites with FITC-labeled SAG1-specific IgY Antibody

The released tachyzoites from the L929 cells were fixed with methanol onto microscopic slides. An FITC-labeled IgY antibody solution was added to the slides, and they were incubated in the dark at room temperature for 30 min. A fluorescence microscope (INV100-FL; BEL Engineering®, Milan, Italy) was used for the imaging interaction between parasites and the FITC-labeled SAG1-specific IgY antibody.

### Staining of *T*. *gondii* Parasites with FITC-labeled IgY Antibody Obtained from the Control Eggs

The methanol-fixed tachyzoites on the microscope slides were added to the FITC-labeled control IgY antibody solution. After incubation in the dark at room temperature for 30 min, the slides were observed under a fluorescence microscope.

### Staining of *T*. *gondii* Parasites with FITC

This experiment was conducted to show whether there is an unspecific interaction between the FITC dye and tachyzoites. For the next step, 400 µl of 10-µg/ml FITC and 0.1 M ethanol amine (Sigma-Aldrich) were mixed at room temperature and added to a thin layer of tachyzoites on a microscopic slide. A fluorescence microscope (INV100-FL; BEL Engineering®, Milan, Italy) was used for the imaging interaction between the tachyzoites and the FITC.

## Results and Discussion

**Figure 1:**
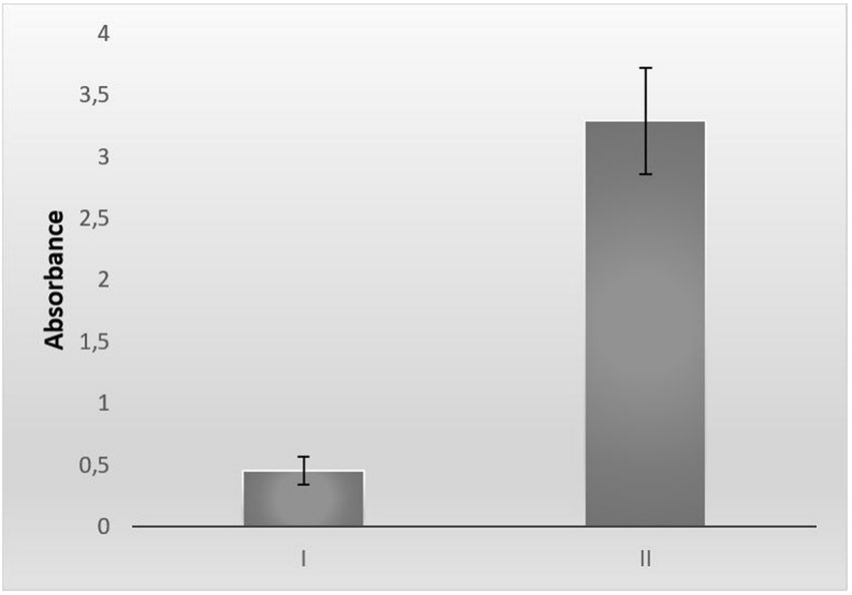
Absorbance values of ELISA. I: The anti-SAG1 IgY antibody amount of eggs in the control group. II: The anti-SAG1 IgY antibody amount of immunized chicken eggs with SAG1.

ELISA was used to show the specificity of the obtained IgY antibody for the SAG1 protein by comparing it with IgY obtained from control hens. As shown in Fig. 1, the absorbance value of antibodies from the control eggs was 0.4, while the absorbance value of the eggs from immunized chickens was nearly 3.2. These results indicated that the IgY antibodies specific to SAG1 were obtained and purified from the immunized hens. There is a specific type of binding that takes place between the SAG1 protein and IgY antibodies from immunized hens. This obtained anti-SAG1 IgY antibody was used for FITC-labeling studies after the amount of protein was detected.

Because the amount of antibodies is an important parameter for the optimization and standardization of fluorescence labeling, the protein amount of anti-SAG1 IgY antibodies was measured using a BioSpec-nano small-volume ultraviolet (UV) spectrophotometer at 280 nm. The absorbance values of the diluted protein solution are given in Table 1. The optical density (OD) values of the diluted protein solution were between 0.2–1.4. The data of the diluted protein solution were obtained as a result of measurements produced by the BioSpec-nano small-volume UV spectrophotometer.

**Table 1:** Absorbance values of specific IgY antibodies obtained from the eggs of immunized hens.

Number of measurement	OD	Molarity **(**10^−5^ **)**	Concentration of protein **(**mg/ml**)**
1^st^	1.213	1.213	2.2
2^nd^	1.192	1.192	2.15
3^rd^	1.224	1.224	2.2

The protein concentrations were determined as the mean value of three measurement results, which was approximately 2.2 mg/ml. The final protein concentration was calculated by using 25 as the dilution coefficient. As a result, the anti-SAG1 IgY antibody concentration was calculated as 54.575 mg/ml. Appropriate dilutions were made according to the concentration detection results.

**Figure 2:**
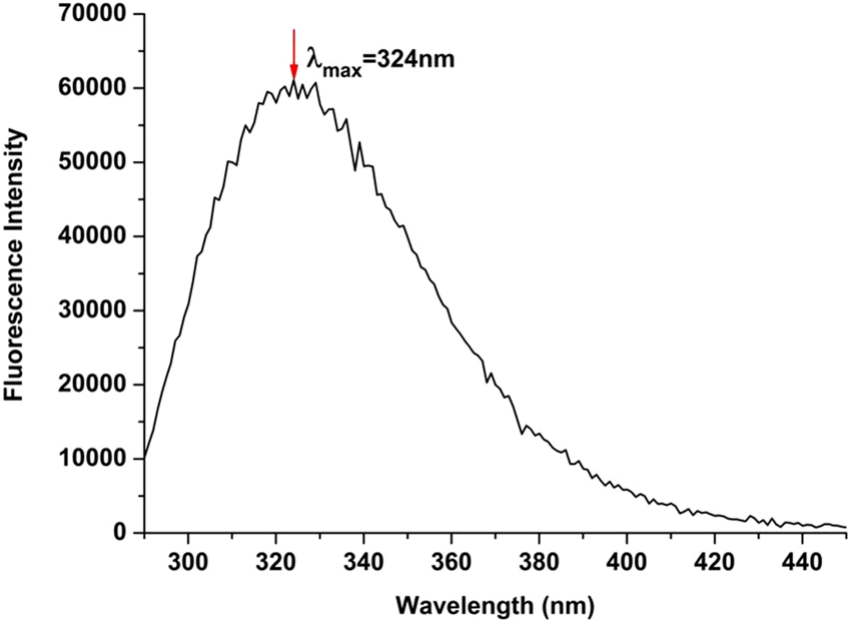
Fluorescence spectra of anti-SAG1 IgY antibody (λ_ex_ = 280 nm).

The typical wavelength of anti-SAG1 IgY antibody showed that the maximum intensity was detected before the FITC labeling studies. As shown in Fig. 2, the maximum fluorescence intensity of the anti-SAG1 IgY antibody was detected at 324 nm.

**Figure 3:**
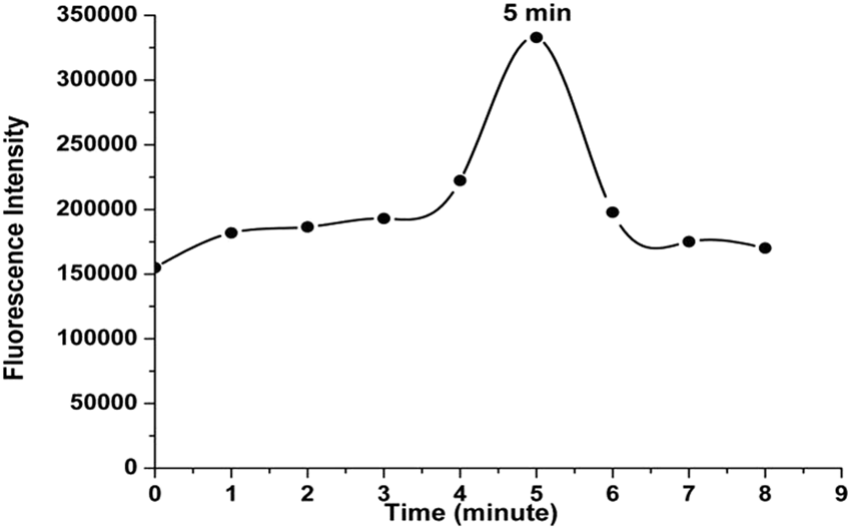
The fluorescence intensity of FITC-added anti-SAG1 IgY antibody against time (λ_ex_ = 495 nm, λ_em_ = 520 nm).

In the IgY antibody and FITC-labeling experiments, the different incubation times were first examined to define the optimum time required for the interaction between the reactive FITC dye and the primary amine groups of the IgY antibody. Figure 3 shows the fluorescence intensity over time, which increased until five minutes had passed; no additional increases were detected after this time. During this period, entire FITC molecules probably interacted with the primary amine groups of the IgY antibody. As a result, the optimum incubation time was five minutes at room temperature in the dark compartment of a fluorescence spectrometer, as noted in the literature^16^ (Fig. 3).

**Figure 4:**
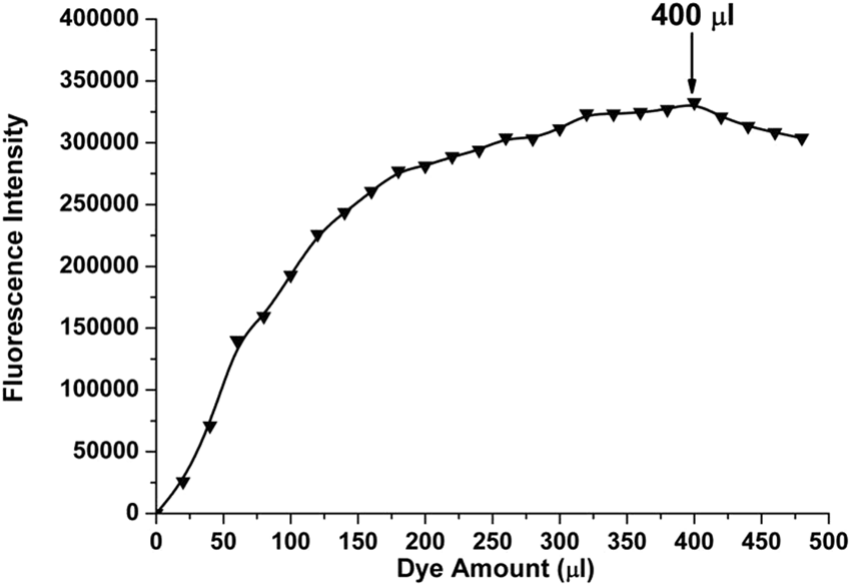
The fluorescence intensity of FITC-added anti-SAG1 IgY antibody against increasing amounts of FITC dye (λ_ex_ = 495 nm, λ_em_ = 520 nm).

The following experiments were conducted to examine the effect of increasing concentrations of FITC on the fluorescence intensity while the amount of IgY antibody remained constant. As shown in Fig. 4, the maximum fluorescence intensity was 400 µl of FITC; additional FITC did not increase the density value because the IgY antibodies were probably saturated with the 400 µl of FITC. Therefore, the optimum FITC concentration and incubation times were determined for labeling specific IgY antibodies against SAG1 with FITC.

FITC is the most popular amine-reactive fluorophore that is commonly used for fluorescence labeling of antibodies. FITC can bind with the side-chain of amino acids, including primary amines as lysines in the structure of the antibody. Antibodies have many different functional groups, but only their primary amine groups are capable of reacting with FITC. In our study, IgY did not appear on the fluorescence spectrum at 520 nm because IgY has a maximum fluorescence intensity of 324 nm (Fig. 2). After the addition of FITC to the IgY solution, FITC reacted with the primary amine groups of IgY and FITC-labeled IgY showed fluorescence emission at 520 nm, which is the characteristic emission wavelength of FITC.

**Figure 5:**
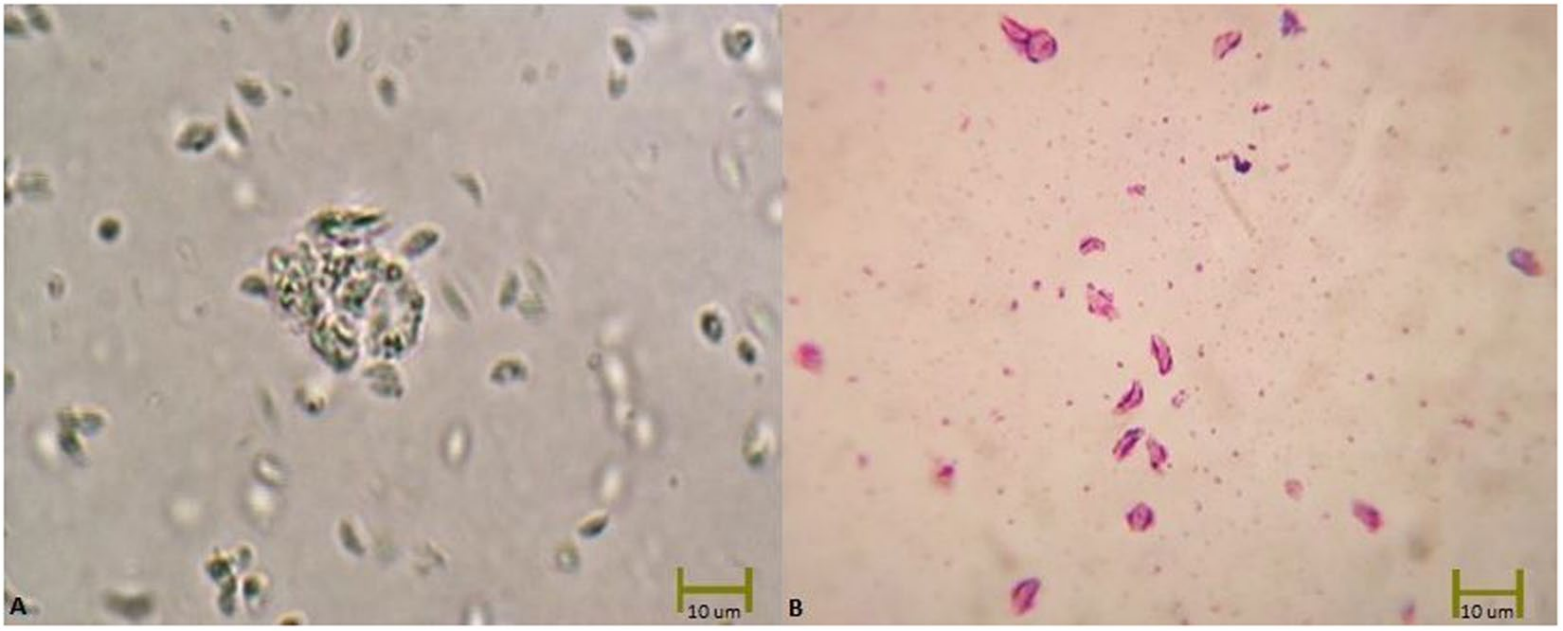
Microscopic images of *T*. *gondii* tachyzoites (**A**). Unstained (x40) (**B**). Stained with Giemsa (x40).

For the cell-imaging experiments, L929 cells were used as the host for cultivation of *T*. *gondii* tachyzoites. The presence of *T*. *gondii* tachyzoites was indicated by both the Giemsa-stained preparations (Fig. 5A) and also those without staining (Fig. 5B) under light microscopy.

**Figure 6:**
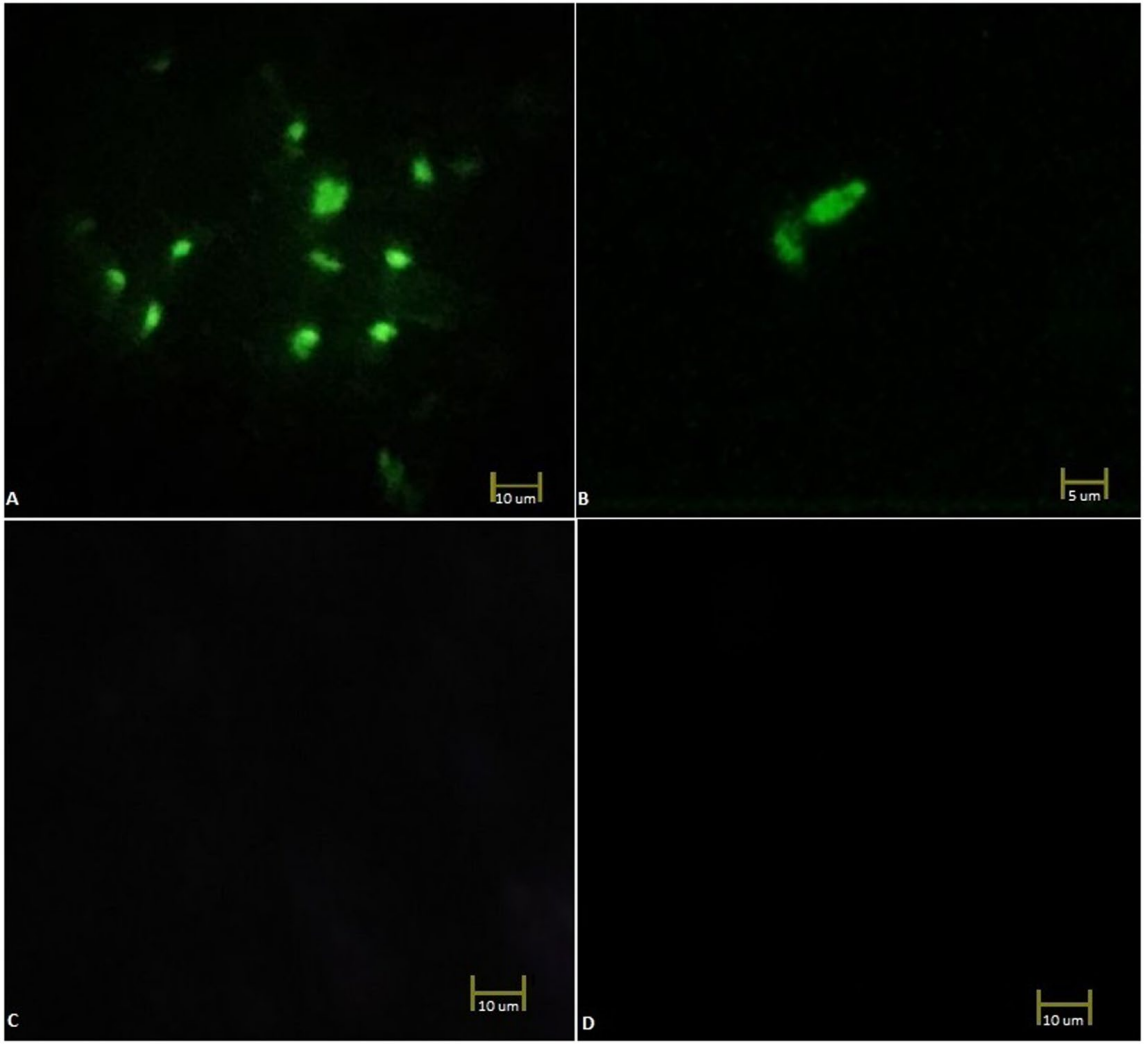
Microscopic images *T*. *gondii* tachyzoites (x40) stained with (**A**,**B**). FITC-conjugated anti-SAG1 IgY antibody, (**C**). FITC-labeled IgY Antibody Obtained from the Control Eggs, (**D**). and FITC (without antibody).

The interaction of the FITC (alone) and FITC-labeled IgY antibodies (SAG1-specific and control) with *T*. *gondii* tachyzoites was examined under a fluorescence microscope. Figure 6 reveals that the FITC-labeled SAG1-specific IgY antibodies specifically reacted with *T*. *gondii*. Fluorescence signals were absent in both the tachyzoites with FITC-labeled control IgY and tachyzoites with FITC dye (Fig. 6C and D). As a result of the fluorescent microscopic examination, only FITC-labeled, SAG1-specific IgY antibodies interacted with *T*. *gondii*, and there were no non-specific interactions between *T*. *gondii* and the FITC-labeled control eggs or FITC alone. Because SAG1 is a surface protein of *T*. *gondii* tachyzoites, the fluorescence-labeled antibodies specific to SAG1 covered the surface of the parasites, and the fluorescence images were compatible with the natural shape of the parasites (Fig. 6).

## Conclusion

In this study, for the first time in the literature, FITC-labeled anti-SAG1 IgY antibody was used to detect *T*. *gondii* tachyzoites *in vitro*, and parameters including the concentration of the dye and the incubation time were optimized for the IgY-labeling experiments.

The detection of specific antibodies in serum samples using serological assays is generally used for the diagnosis of toxoplasmosis^17^. However, directly identifying the presence of a parasite is very important because it is frequently difficult to define whether the infection was recent or old using only the detected antibodies^18^ in addition, antibody detection methods cannot differentiate between vaccine-induced and infection-induced antibodies^17^. While the polymerase chain reaction method can also be used to show the presence of parasites, it cannot distinguish between viable intact and non-viable, fragmented parasites^19^. Therefore, the direct detection of parasites using different methods, such as fluorescent-labeled antibodies, retains its importance in clinical practice.

Our results showed that fluorescent-labeled SAG1 antibodies interacted with the tachyzoites and produced fluorescent images. Until recently, tachyzoites were not considered important sources of infection because they are sensitive to the proteolytic enzymes of milk and gastric juices and usually break down. However, previous studies have shown that tachyzoites can survive in a solution with pepsin, and orally administered tachyzoites could infect cats^20, 21^. Under these circumstances, the detection of *T*. *gondii* tachyzoites gains further importance, and the fluorescence-labeled antibody against *T*. *gondii* tachyzoites developed in this study may be useful for the identification of *T*. *gondii* tachyzoites in humans, animals, foods, and water. SAG1 is a tachyzoite-specific antigen of and is highly conserved between strains and is therefore indicated as a good candidate for diagnosis^22^. Some studies also showed that the rSAG1 does not have any cross reactivity against proteins of other microorganisms^23^.

This study showed the potential of the developed antibody-FITC conjugate for the detection of tachyzoites in different sources, such as milk, contaminated water, and body fluids. In the future, this FITC-labeled SAG1-specific IgY antibody may be commercially used for diagnostic purposes after extensive cross reactivity, validation, and specificity tests have been conducted with different samples.
